# Microbial synthesized biodegradable PHBHHxPEG hybrid copolymer as an efficient intracellular delivery nanocarrier for kinase inhibitor

**DOI:** 10.1186/1472-6750-14-4

**Published:** 2014-01-18

**Authors:** Xiao-Yun Lu, Ming-Chuan Li, Xin-Liang Zhu, Fan Fan, Lei-Lei Wang, Jian-Gang Ma

**Affiliations:** 1 Department of Biological Science and Bioengineering, Key Laboratory of Biomedical Information Engineering of Ministry of Education, School of Life Science and Technology, Xi’an Jiaotong University, Xi’an 710049, Shaanxi, P. R China; 2 Molecular Biotechnology Center, Universita di Torino, 10126 Torino, Italy

**Keywords:** Polyhydroxyalkanoate, PEG, Rapamycin, Nanoparticle, Drug delivery

## Abstract

**Background:**

Protein Kinases are key regulators of cell function and play essential roles in the occurrence and development of many human diseases. Many kinase inhibitors have been used for molecular targeted treatment of those diseases such as cancer and inflammation. However, those highly hydrophobic kinase inhibitors shared the common features of poor bioavailability and limited in vivo half-life, which strongly impeded their practical applications. Our previous study demonstrated that microbial synthesized biodegradable polyester poly(3-hydroxybutyrate-*co*-3-hydroxyhexanoate) (PHBHHx), a member of polyhydroxyalkanoates (PHAs) family, could serve as a promising delivery nanocarrier for those hydrophobic kinase inhibitors. Recently, a novel natural synthesized hybrid copolymer, PEG200 end-capped PHBHHx (PHBHHxPEG) was produced by *Aeromonas hydrophila* fermentation. In this study, the novel PHBHHxPEG NPs were prepared and investigated to serve as intracellular delivery nanocarriers for sustained release of hydrophobic kinase inhibitors.

**Results:**

PHBHHxPEG nanoparticles (NPs) prepared by an emulsification–solvent evaporation method were spherical with a diameter around 200 nm. The entrapment efficiency on rapamycin in PHBHHxPEG NPs was 91.9% and the sustained release of rapamycin from PHBHHxPEG NPs could be achieved for almost 10 days. The cellular uptake of PHBHHxPEG NPs was significant higher than that of PHBHHx NPs. The anti-proliferation effect and mTOR inhibition ability of rapamycin-loaded PHBHHxPEG NPs was stronger than that of drug-loaded PHBHHx NPs and free rapamycin.

**Conclusions:**

PHBHHxPEG NPs could achieve the efficient entrapment and sustained release of rapamycin. The novel biodegradable PHBHHxPEG appeared a promising nanocarrier for sustained delivery of hydrophobic kinase inhibitors with improved cellular uptake and kinase inhibition efficiency.

## Background

Protein kinases play essential roles in signal transduction and regulation by phosphorylating other proteins to alter their enzymatic activity, cellular location, or association with other proteins. Phosphorylation of the target protein is a necessary and key step in the occurrence and development of some cancers and inflammatory diseases. Therefore, protein kinase inhibitors which specifically block the action of one or more protein kinases have been used as drugs for molecular targeted treatment of cancer and inflammatory diseases. However, poor bioavailability and limited in vivo half-life are rather common features of many kinase inhibitors that usually are highly hydrophobic compounds. For example, rapamycin (RAP, also known as Sirolimus) isolated from *Streptomyces hygroscopicus* is such kind of hydrophobic kinase inhibitor which has already been approved by US Food and Drug Administration (FDA) as an immunosuppressive drug to prevent the rejection of solid organ transplants [1]. RAP is a natural inhibitor of the mammalian target of rapamycin (mTOR) which is a critical regulator of cell growth, proliferation and survival. Dysregulation of mTOR activities often occurs in a variety of human malignant diseases, making it a crucial and validated target for the cancer intervention [2]. The anti-proliferation activity of RAP was evaluated and it was found that RAP could also inhibit the proliferation of a number of tumor cell lines [3]. However, since it is a strongly hydrophobic compound and only slightly soluble in several acceptable excipients such as ethanol, propylene glycol and polyethylene glycol 400 [4], RAP is only available in oral but with a relative low bioavailability of around 17% [5]. Therefore, conventional dosage formulations of RAP could not fulfill the demand for anti-proliferation therapy.

Biodegradable polymeric nanoparticles are more and more frequently used in drug delivery systems and represent one of the most rapidly developing areas, which have now attracted growing interest of chemists, pharmacists and biologists. Nanosized polymeric nanoparticles provide a comprehensive platform for achieving the enhanced drug solubility/stability, improving the effectiveness of drug therapy and reducing the side effects of the loaded drug to healthy tissues. Many different natural and synthetic polymers have been developed as drug delivery carriers. Polyhydroxyalkanoates (PHAs) are a family of biodegradable aliphatic polyesters which are synthesized by a wide range of bacteria. PHAs have been extensively studied as implantable tissue repair/regeneration devices and other biomedical devices such as sutures and suture fasteners, because of their good biocompatibility and biodegradability [6,7]. Recently, PHAs have also been explored for controlled drug-release applications [8,9]. Based on their polyester aliphatic properties, PHAs are more efficient in encapsulating the hydrophobic compounds and thus would be especially beneficial for hydrophobic drugs. Several commercialized PHAs, for example, polyhydroxybutyrate (PHB) and poly(3-hydroxybutyrate-*co*-3-hydroxy-hexanoate) (PHBHHx), have been successfully developed into nanoparticles and investigated for the entrapment and release profiles of hydrophobic compounds. The drug-loaded PHA nanoparticles demonstrated sustained release of hydrophobic drugs and enhanced bioavailability in cell-based assays [8].

In our previous studies, a novel naturally synthesized hybrid polyester, polyethylene glycol 200 (PEG200) end-capped poly(3-hydroxybutyrate-*co*-3- hydroxyhexanoate) (PHBHHxPEG) was directly produced by *Aeromonas hydrophila* fermentation [10]. In this study, the novel PHBHHxPEG NPs were prepared and investigated to serve as the nanocarrier for sustained release of a hydrophobic kinase inhibitor like RAP. RAP cellular uptake of PHBHHxPEG NPs, *in vitro* release of RAP from the NPs, cellular proliferation inhibition and kinase inhibition of RAP-loaded NPs were investigated. The results obtained in this study indicated that the microbial synthesized PHBHHxPEG hybrid copolymer represents a promising material to serve as an intracellular drug delivery carrier for sustained release of hydrophobic kinase inhibitors.

## Results

### Preparation and characterization of PHBHHx and PHBHHxPEG NPs

**Figure 1:**
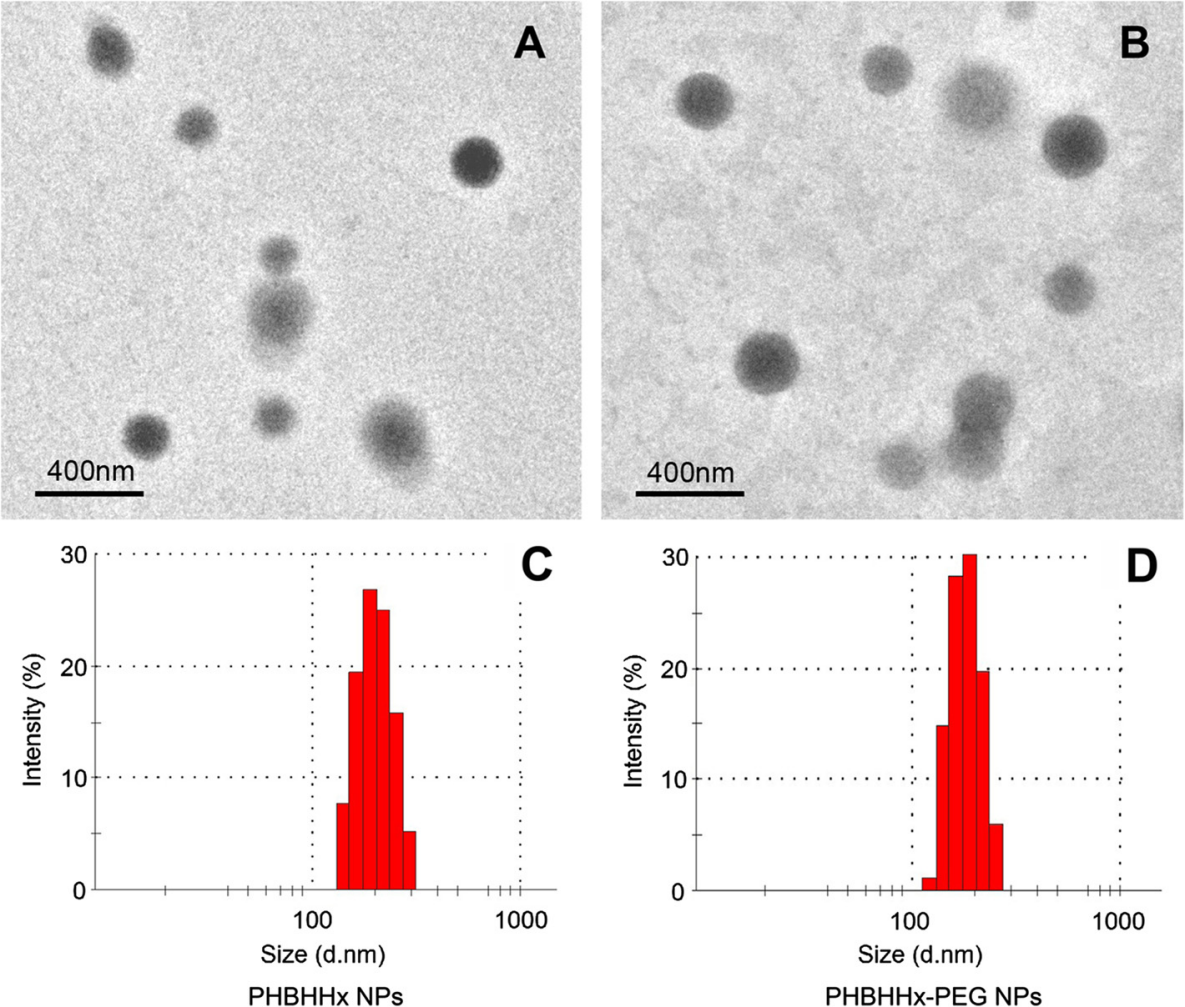
TEM photographs (A, B) and the NPs size distribution (C, D) of PHBHHx NPs (A, C) and PHBHHx-PEG NPs (B, D).

Two kinds of free nanoparticles (PHBHHx and PHBHHxPEG), two kinds of rhodamine-loaded nanoparticles (PHBHHx and PHBHHxPEG) and three kinds of rapamycin-loaded nanoparticles (PLA, PHBHHx and PHBHHxPEG) were prepared by an emulsification–solvent evaporation technique. The sizes and polydispersities of the nanoparticles were listed in Table 1. Figure 1 showed the TEM photographs and the size distribution of PHBHHx and PHBHHxPEG NPs, respectively. The sizes of nanoparticles ranged from 100 to 300 nm after 6 h stirring with relative low polydispersity index (PDI). At the drug/material feeding ratio of 1:10, entrapment efficiency (EE) of rapamycin in both PHBHHx and PHBHHxPEG NPs were higher than 90%. The drug loading content (DLC) of RAP-loaded PHBHHx and PHBHHxPEG NPs were 8.52% and 8.47%, respectively. PLA NPs demonstrated lower entrapment ability compared with PHBHHx and PHBHHxPEG NPs, which was consisted with our previous report.

**Table 1:** The diameters and polydispersities of the free and rapamycin/rhodamine-loaded nanoparticles

**NPs**	**D**_**mean**_**±SD (nm)**	**PDI**	**EE±SD (%)**	**DLC±SD (%)**
PHBHHx	196.2±39.6	0.023	—	—
PHBHHxPEG	178.3±31.5	0.045	—	—
PLA-rapamycin	203.0±37.2	0.025	64.1±2.2	6.01±0.3
PHBHHx-rapamycin	211.4±36.5	0.048	92.7±3.4	8.52±0.7
PHBHHxPEG-rapamycin	206.7±33.1	0.044	91.9±3.8	8.47±0.5
PHBHHx-rhodamine	202.7±42.9	0.091	—	—
PHBHHxPEG-rhodamine	202.2±34.2	0.018	—	—

*Abbreviations:NPs* nanoparticles, *D*_*mean*_, mean diameter, *SD* standard deviation, *PDI* polydispersity index, *EE* Entrapment efficiency.

### In vitro release of rapamycin

**Figure 2:**
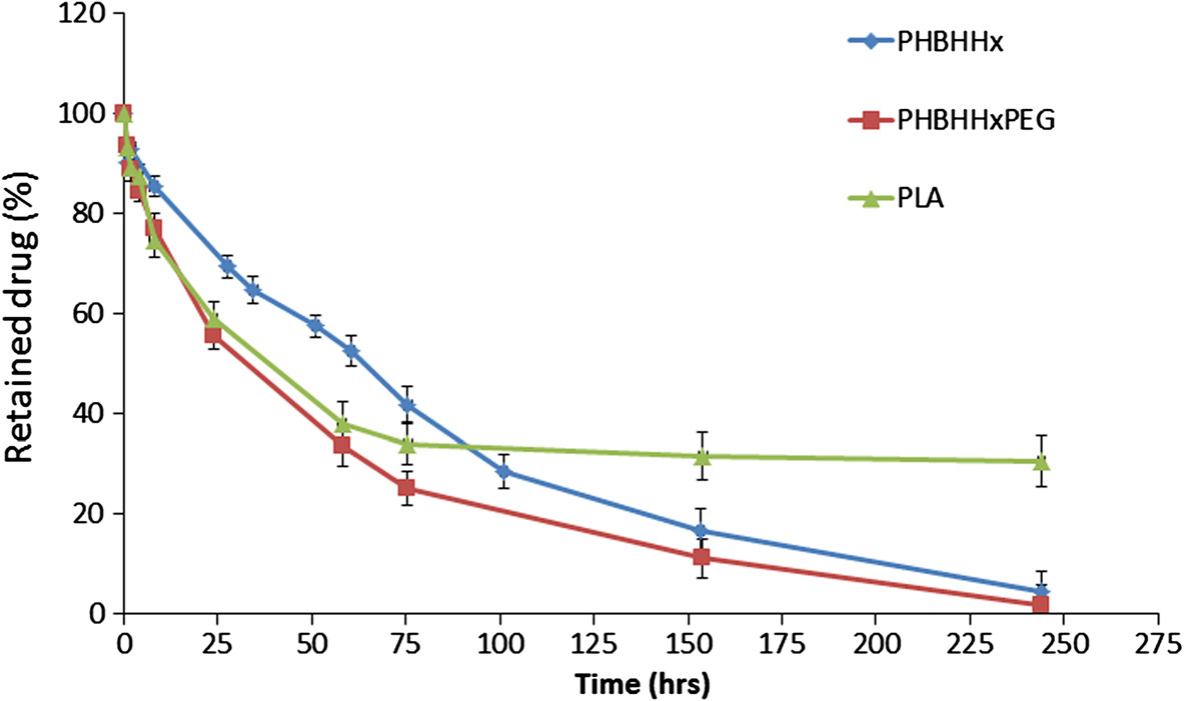
In vitro release profiles of RAP-loaded PHBHHx, PHBHHxPEG and PLA nanoparticles.

Three kinds of RAP-loaded NPs were prepared based on PLA, PHBHHx and PHBHHxPEG, respectively. Figure 2 shows the *in vitro* release profiles of RAP-loaded NPs in water at 37°C. PLA and PHBHHxPEG NPs showed similar profiles of an initial burst release followed by a sustained discharge stage, which was a little bit faster than that from PHBHHx NPs. About 40% of the drug could be released within the first 24 hrs from PLA NPs and PHBHHx-PEG NPs and only about 30% of RAP was released from PHBHHx NPs during the same period. At 75 hours, the percentage of retained drug in PHBHHx and PHBHHxPEG NPs were 42% and 25%, respectively, which demonstrated that the release of RAP from PHBHHxPEG NPs was faster than that from PHBHHx NPs.

Interestingly, the release of RAP from PHA-based NPs was more efficient than that from PLA NPs. Indeed, more than 95% of the drug could be released from the PHBHHx and PHBHHxPEG NPs after 10 days. On the contrary, more than 30% RAP still remained in the PLA NPs even after 10 days, which indicated that this 30% of drug was strongly encapsulated in the PLA NPs and could not be released. Such behavior might be explained by the relativly poor crystalline nature of PHBHHx and PHBHHxPEG materials, thus allowing RAP to diffuse from the loose particle core. These results thus indicated that the PHBHHx and PHBHHxPEG nanoparticles could efficiently and effectively extend the release profile of RAP and could be eventually used as controlled delivery carriers for hydrophobic drugs.

### Cellular uptake of PHBHHx and PHBHHxPEG NPs

**Figure 3:**
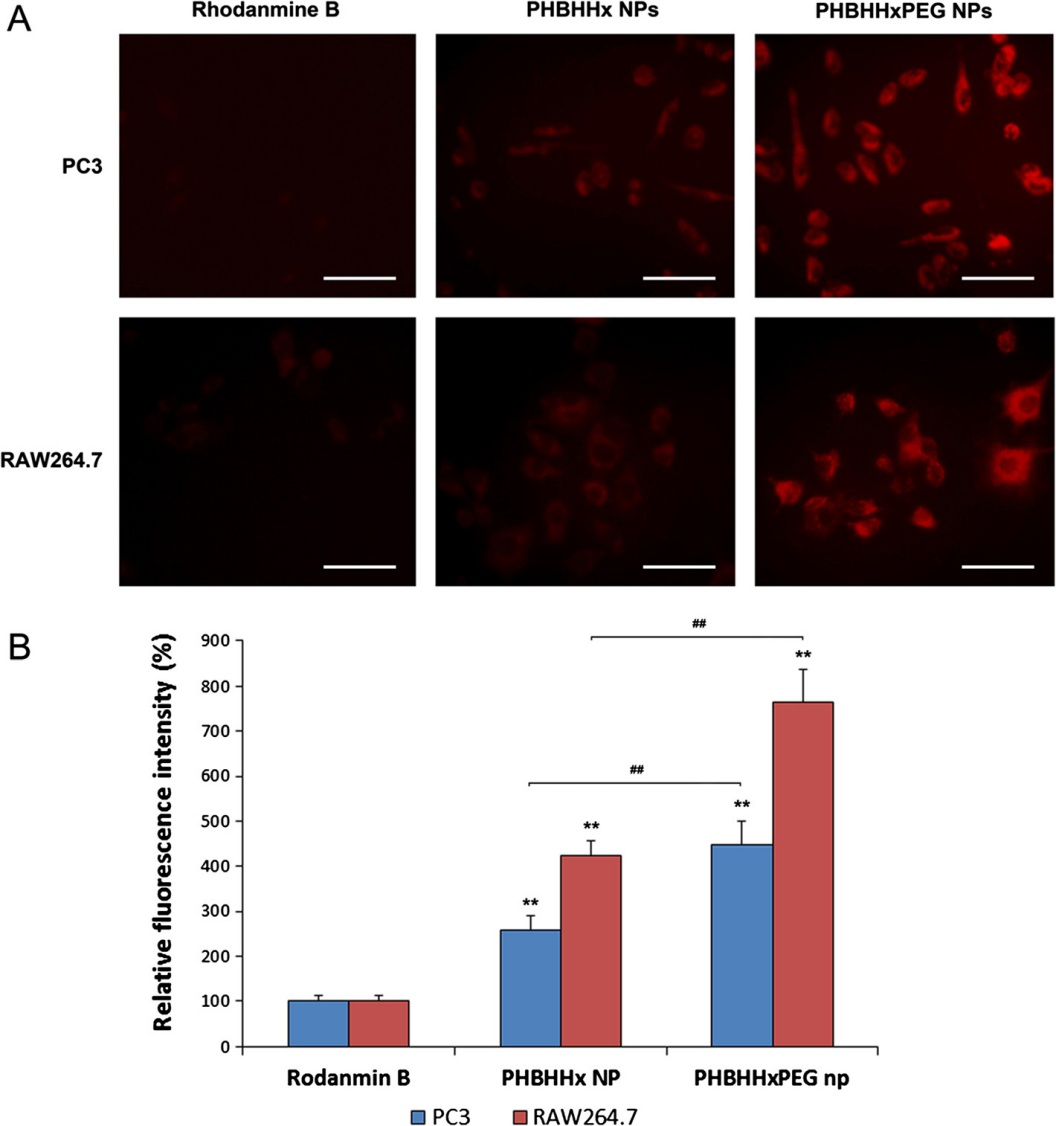
Uptake of PHBHHx and PHBHHxPEG nanoparticles by PC3 and RAW264.7 cells. A: Fluorescence microscopy pictures; B: Intracellular fluorescence intensities quantification by fluorescence spectrophotometer.

Human prostate cancer cell line PC3 and a murine macrophage cell line RAW264.7 were used to investigate the *in vitro* endocytosis of PHA-based NPs. Cells were treated with the rhodamine B solution, rhodamine B-loaded PHBHHx NPs and PHBHHxPEG NPs in the same fluorescence intensity, respectively. After 3 hrs treatment, the intracellular fluorescence of the rhodamine B solution treated group was weak in both PC3 and RAW264.7 cells, which suggested that rhodamine B could not be easily absorbed by both these two cell lines (Figure 3A). However, significant intracellular fluorescence signal could be detected either by the fluorescence microscopy observation (Figure 3A) or by fluorescence intensity analysis (Figure 3B) in both PC3 and RAW264.7 cells 3 hrs after the addition of rhodamine B–loaded NPs. The internalized NPs were mainly accumulated in the cytoplasm and didn’t reach the cell nucleus area, indicated by the weak fluorescence intensity area inside the cells. Moreover, the rhodamine B–loaded PHBHHxPEG NPs treating group showed much stronger intracellular fluorescence intensity compared to the rhodamine B–loaded PHBHHx NPs treating group, indicating that PHBHHxPEG NPs showed higher cell affinity and easier cellular uptake.

### In vitro cytotoxicity evaluation of PHBHHx and PHBHHxPEG NPs

**Figure 4:**
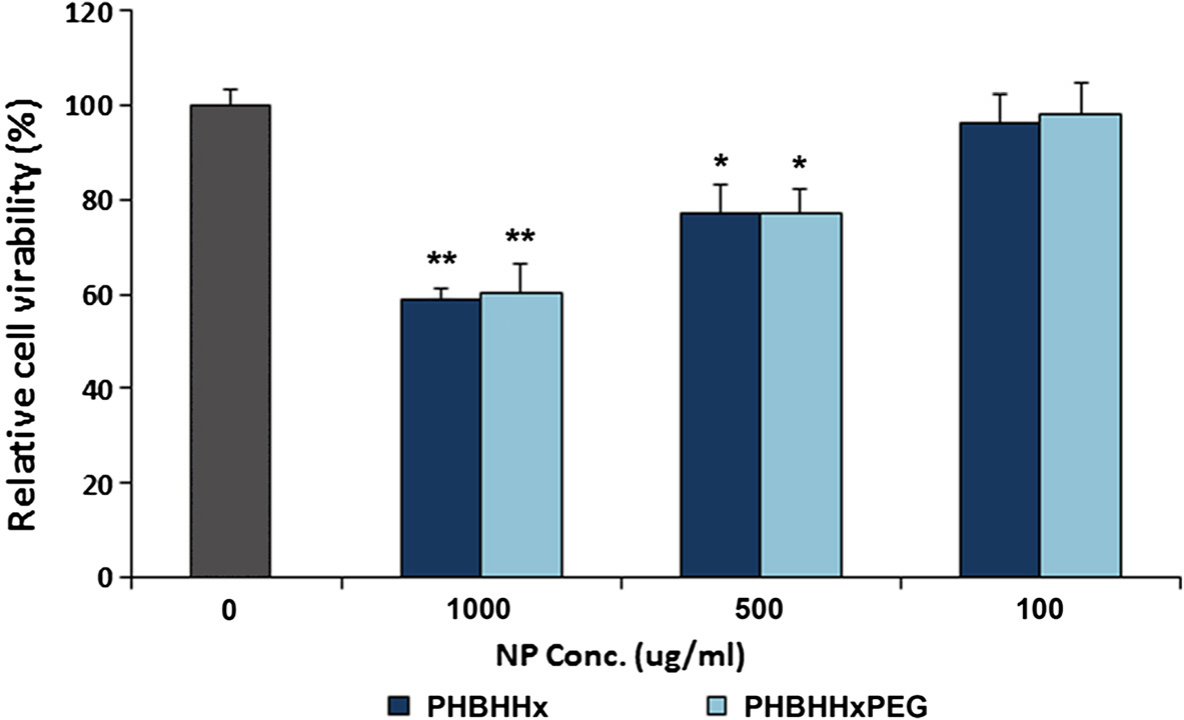
In vitro cytotoxicity of PHBHHx and PHBHHxPEG NPs on PC3.

The cytotoxic effect of PHBHHx and PHBHHxPEG NPs on PC3 was evaluated *in vitro* by MTT assay. Figure 4 shows relative proliferation percentage of PC3 incubated with different NPs at doses of 100, 500 and 1000 μg ml^-1^ for 24 h, respectively. PHBHHx and PHBHHxPEG NPs could be well tolerated at the concentration of 100 μg ml^-1^. However, decreased cell viability was observed in the groups treated with 1000 μg ml^-1^ and 500 μg ml^-1^ NPs, which resulted in 40% and 20% decrease of cell viability, respectively. PHBHHx NPs and PHBHHxPEG NPs-treated groups did not show significant difference in cell viability.

### Effects of rapamycin-loaded NPs on proliferation of PC3 cells

**Figure 5:**
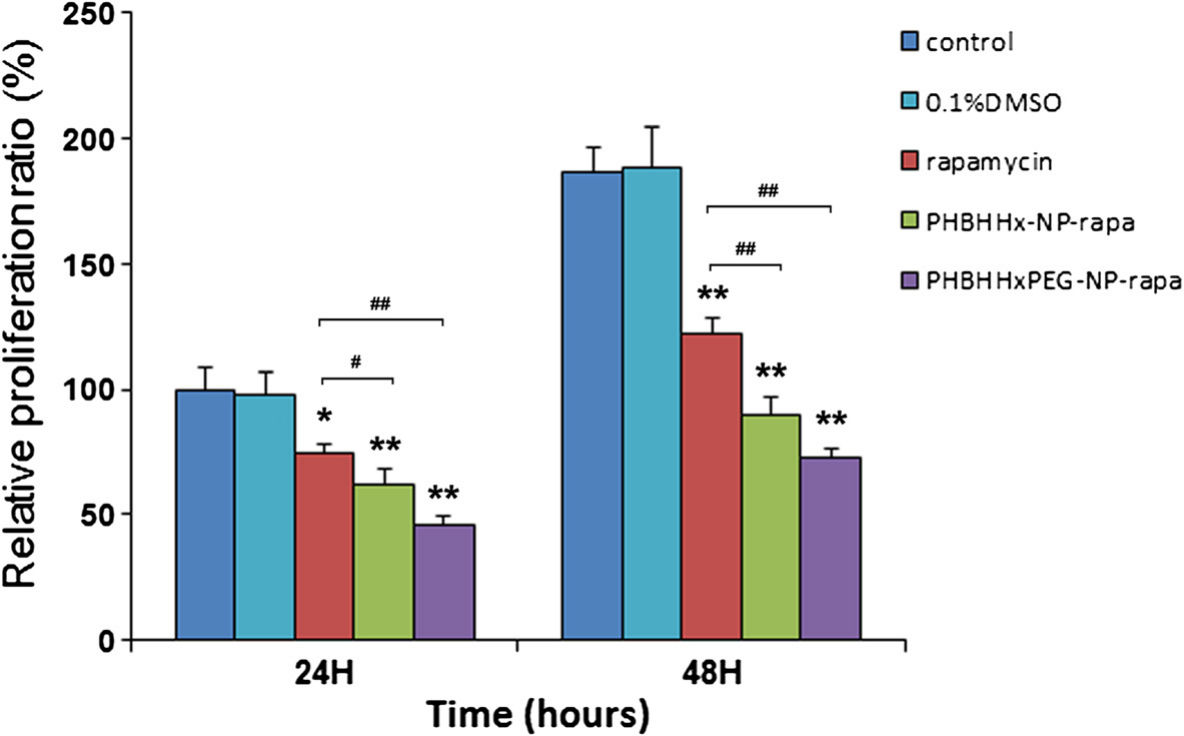
The effect of RAP-loaded NPs on the proliferation of PC3.

The anti-proliferation effect of RAP-loaded NPs on PC3 cells was evaluated by the MTT assay. Cells were treated with 10 μM free RAP, RAP-loaded PHBHHx (100 μg ml^-1^) and PHBHHxPEG NPs (100 μg ml^-1^) which contained equal amounts of drug, respectively, for 48 h. PC3 cells showed a dramatic inhibition of cellular proliferation when treated with RAP-loaded NPs compared to cells treated with free RAP at the same concentration (Figure 5). The anti-proliferative effects persisted after 48 hours without further addition of NPs, potentially because of the sustained intracellular release of RAP from the NPs. In line with the efficient cellular uptake and the fast voiding rate of PHBHHxPEG NPs, RAP-loaded PHBHHxPEG NPs inhibited PC3 cell proliferation more efficiently than RAP-loaded PHBHHx NPs.

### Effects of RAP-loaded NPs on mTOR activity

**Figure 6:**
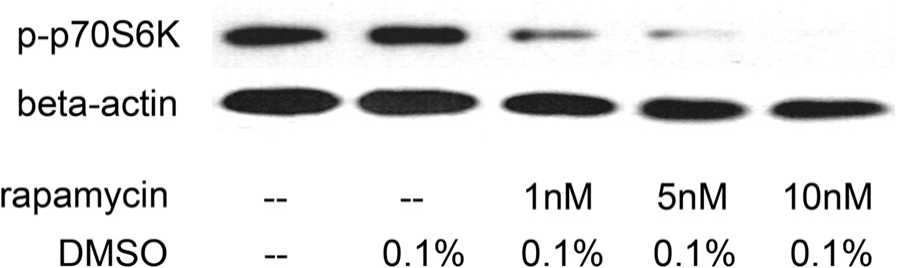
Analysis of p70 S6K phosphorylation after treated with different concentrations of RAP.

**Figure 7:**
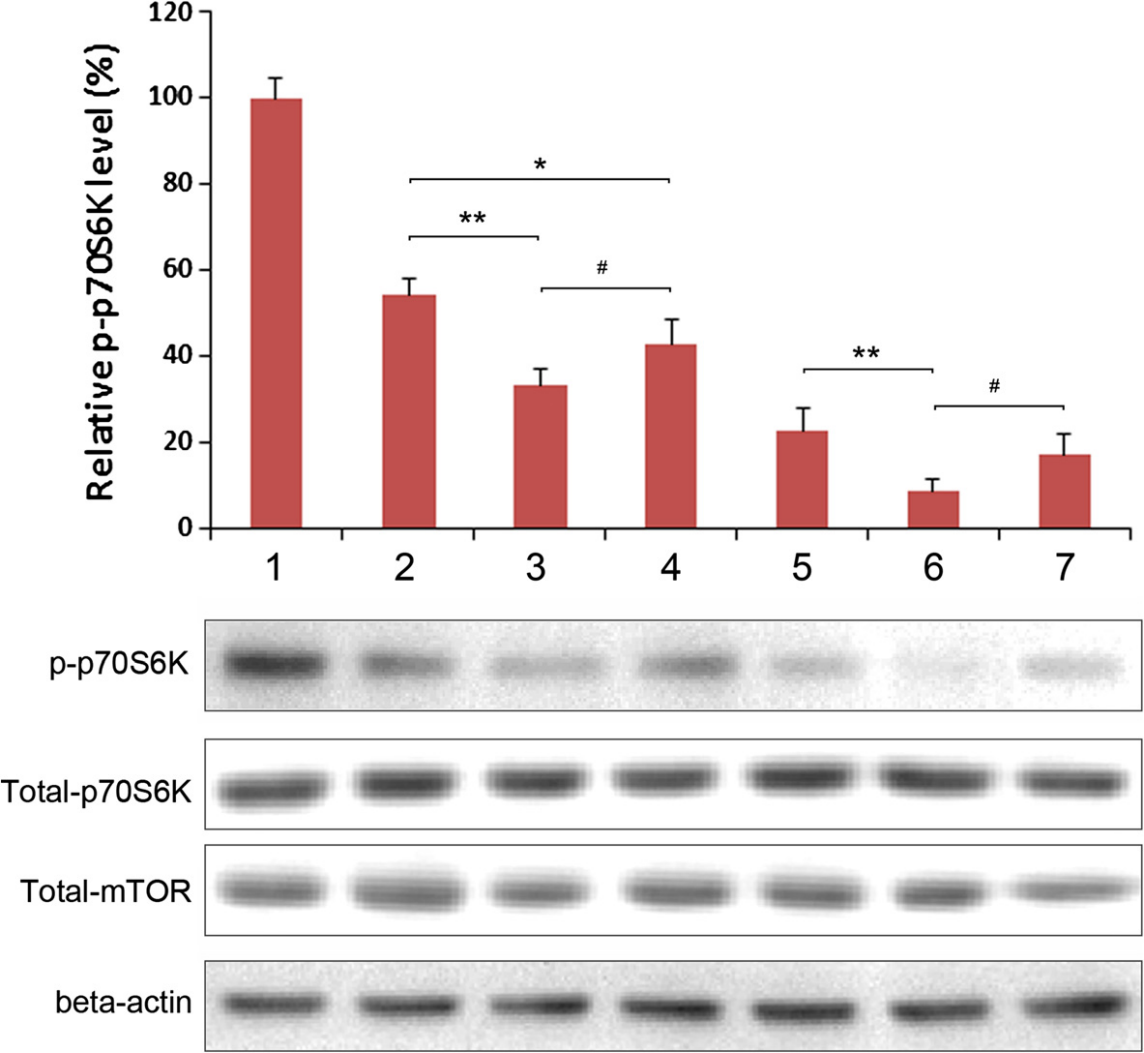
Analysis of p70 S6K phosphorylation after treated with different concentrations of RAP-loaded NPs.

p70S6K (p70 S6 ribosomal protein kinase) is a main downstream mediator of mTOR and phosphorylation level of p70S6K at Thr389 could be used as a probe for mTOR activity. Different from the anti-proliferation effect on PC3, phospho-p70S6K signaling could be significant inhibited by RAP at a very low concentration (Figure 6), as previosly reported [11,12]. The inhibition on mTOR activity in PC3 could also be achieved by adjusting drug-loaded NPs dosage (10–50 ng ml^-1^) to reach a final RAP concentration of either 1 or 5 nM (Figure 7). Treatment with free RAP, RAP-loaded PHBHHxPEG NPs and RAP-loaded PHBHHxPEG NPs equally resulted in decreased p70S6K phosphorylation (Thr389), without affecting total p70S6K and total mTOR levels. This indicated that the decreased phosphorylation level of p70S6K was only due to the inhibition of mTOR activity but not because of the downregulation of mTOR or p70S6K. In keeping with their anti-proliferative effects, drug-loaded PHBHHx and PHBHHxPEG NPs demonstrated to better inhibit mTOR than free RAP. RAP-loaded PHBHHxPEG NPs showed even stronger inhibition effects than RAP-loaded PHBHHx NPs.

## Discussion

Because poor bioavailability and limited *in vivo* half-life are rather common features of many kinase inhibitors that usually are highly hydrophobic compounds, the development of polymer-based nanocarrier for kinase inhibitors provided a promising strategy to improve their therapeutic activity by releasing drugs in a sustained manner, and consequently enhancing their bioavailability, improving their solubility, and reducing the toxic side effects [13]. In addition, drug-carrying nanoparticles could be passively accumulated in tumor or inflammatory tissues through the enhanced permeability and retention (EPR) effect and thus could further increase the effectual concentration of hydrophobic drugs [14]. Polymeric nanoparticles have been extensively studied as drug carriers [15] and several polymer-based, nanoparticulated formulations have been approved by the FDA for clinical applications [16]. PHAs are linear biopolyesters produced as energy- and carbon-storage materials by many bacteria. The medical applications of PHA have been extensively explored in recent years for implant biomedical applications [6]. Several members of PHAs, such as PHB and PHBHHx, have also been developed into nanoparticles and investigated for controlled drug-release applications [8,9,17]. Our previous data indicated that they were efficient in encapsulating hydrophobic compounds such as the phosphoinositide-3-kinases (PI3Ks) inhibitor TGX221 (Mw = 364.4). In this study, rapamycin, a mTOR inhibitor with relative high molecular mass of 914.17, was used to investigate the entrapment and release abilities of PHA NPs for high molecular weight kinase inhibitors. Both PHBHHx and PHBHHxPEG NPs showed higher rapamycin entrapment efficiency (more than 90%) and more complete drug release than that of PLA NPs, which was consisted with the study of TGX221-loaded PHA NPs. This indicated the advantage of PHA-based NPs in the entrapment and sustained release of hydrophobic kinase inhibitors. The results of this study also showed that release of rapamycin from PHBHHxPEG NPs was faster than that from PHBHHx NPs, which might be due to the incorporation of PEG fragments and the relativly low molecular weight of PHBHHxPEG.

The incorporation of PEG could also confer more hydrophilicity to PHBHHxPEG NPs. This might explain the enhanced cellular uptake of PHBHHxPEG NPs, since a hydrophobic surface is known to disturb cell interaction. Yang et al. reported that the surface of PHA film demonstrated higher hydrophilicity and better cell compatibility after NaOH treatment [18]. This can be explained by the ability of NaOH treatment to break down the ester bond of PHA backbone, leaving more hydroxyl group and carboxyl group on the surface of PHA film. Our previous study also indicated that the NaOH treatment increased the surface hydrophilicity of PHBHHx film and the neural stem cell-PHBHHx film interaction (data not published). The hydrophilic PEG represents a biocompatible material commonly used to enhance the biocompatibility of an object with good cell compatibility. Therefore, the emulsification/solvent evaporation method was used to expose PEG at the surface of PHBHHxPEG NPs and hence to increase the hydrophilicity of NPs surface as well as the cell-NPs interaction. In agreement, our results showed that cell contact was maximal in PHBHHxPEG NPs.

PEGylation of nanocomposites was reported to be able to resist nonspecific protein adsorption and have prolonged circulating half-life *in vivo*[19]. However, in this study, the PHBHHxPEG NPs could not prevent the endocytosis by RAW264.7 murine macrophage-like cells. This might be explained by an insufficient PEG density on the colloidal surface of PHBHHxPEG NPs. PEG-modification of PLGA NPs is known to improve circulation time, depending on the molecular weight (chain length) and molar ratio (grafting efficiency) of PEG incorporation [20]. After 5 min from administration, the remaining PLGA particles in circulation are about 5%, 25% and 50% for unmodified, PEG5000 modified and PEG20000 modified PLGA particles, respectively. This shows that the long PEG chain is necessary for increased anti-clearance properties. In our study, the PHBHHxPEG copolymer was synthesized by *A. hydrophila* by adding PEG200 in the culture medium. PEG200 attacks the carbonyl carbon of the PHBHHx chain directly and forms the end-capped PHBHHxPEG copolymer [10]. Thus, in our experiments the incorporated PEG fragment was no longer than 3–4 units in each chain. While this was insufficient to promote the formation of a dense, hydrophilic cloud on the surface of NPs, potentially preventing macrophage-dependent opsonization, the use of PEG200 was sufficient to optimize NPs hydrophilicity and bioavailability.

## Conclusions

Overall, this study represented the potential of PHBHHxPEG NPs as a promising kinase inhibitor delivery carrier in anti-cancer study. Our results indicated that the entrapment of rapamycin into PHBHHxPEG NPs could sufficiently enhance its bioavailability and anti-proliferation effect. Nonetheless, *in vivo* evaluation of circulating time and anti-proliferation ability of the rapamycin-loaded PHA NPs in animal models is still needed. Conversely, the proven effectiveness in cell based assays of this novel intracellular delivery nanocarrier opens the way to similar formulation of other kinase inhibitors. Since the PHBHHxPEG NPs based drug delivery carrier could also be internalized by other cell types such as macrophage, rapamycin-loaded PHBHHxPEG NPs might also have alternative applications, for example, in the field of immunosuppressant therapeutic tregimens.

## Methods

### Materials

Poly(3-hydroxybutyrate-*co*-3-hydroxyhexanoate) (PHBHHx) (Mw =4 × 10^5^) was donated by Lab of Microbiology, Department of Biological Science and Biotechnology, Tsinghua University (Beijing, P. R. China). PHBHHxPEG (Mw =1.8 × 10^5^) was produced by our group [10]. Poly(vinyl alcohol) (PVA) (P1763), poly(DL-lactide) (PLA) (P1691) and rhodamine B (83689) were purchased from Sigma-Aldrich (USA). Rapamycin (S1039) was purchased from selleckchem (USA).

### Preparation of drug-Loaded PHBHHx-PEG nanoparticles

The RAP-loaded NPs and rhodamine B-loaded NPs were fabricated by a modified emulsification/solvent evaporation method [8,9,21]. Briefly, 20 mg of polymers and RAP (2 mg) or rhodamine B (1 mg) were added into 1 ml chloroform and the mixture was stirred to ensure that all materials were dissolved. Then the polymer-drug organic solution was slowly dropped into 20 ml of 1% PVA (w/v) under sonication. The double emulsion was then deal with sonication using a probe sonicator (Sonics & Materials, Newtown, CT, USA) for 5 min, followed by moderately stirred with a magnetic mixer for 10 h to solidify the nano-droplets. Chloroform was removed by volatilization at room temperature. The NPs were collected by centrifugation at 12000 rpm for 30 min and then washed twice with phosphate buffered saline (PBS) solution. Three types of hydrophobic polymer NPs (PHBHHx, PHBHHxPEG and PLA) were prepared in this study.

### Characterization of nanoparticles

The particle size distribution and polydispersity index (PDI) were measured by a laser light scattering machine (Zetasizer Nano ZS, Malvern, UK). The samples were diluted into proper concentration with deionized water and examined to determine the average particle diameters and PDI. The shape of resulting NPs was observed by JEM-2100 transmission electron microscope (JEOL Ltd. Japan).

### Entrapment efficiency and drug-loading content measurement

The drug entrapment efficiency refers to the amount of RAP loaded into the NPs as compared with the total amount fed at the beginning. The RAP entrapment efficiency and drug loading content (DLC) was calculated according to the equations (1) and (2).

(1) $$\mathrm{Entrapment}\;\mathrm{efficiency}\;\left(\%\right)=\left(M_{t}/M_{i}\right)\times 100\%$$

(2) $$\mathrm{DLC}\left(\%\right)=\left(M_{t}/M_{p}\right)\times 100\%$$

Where M_i_ was the mass of RAP fed initially, M_t_ represented for the total amount of RAP in NPs and M_p_ was the mass of the resulting NPs, respectively [22]. The amount of RAP was analyzed by high performance liquid chromatography (HPLC) with a SinoChrom ODS-BP column (5 μm, 4.6 mm × 15 mm; Elite Analytical Instruments, Dalian, P.R. China). To measure the amount of entrapped or retained RAP in NPs, 50 microliters of resuspended RAP-loaded NPs were dissolved in 450 μl acetonitrile at room temperature and then stored at 4°C for 4 hours to precipitate polymer. The supernatant was used to perform the HPLC analysis after filtrated with 0.2 μm filter.

### In vitro drug release study

*In vitro* release studies of RAP from PHBHHx, PHBHHxPEG and PLA NPs were performed by the dialysis bag method [22]. Briefly, 10 ml NPs suspension was placed in a dialysis membrane bag with a molecular weight cutoff of 8000 ~ 14000 g/mol. The bag was tied and immersed into a 500 ml beaker with sustained running water to ensure the sufficient dissolution of RAP in the water outside the bag. The entire system was kept at 37°C with continuous stirring. Then 50 microliters of the NPs solution was taken out from the dialysis bag at predetermined time intervals. The amount of retained RAP (Cr) and the initial amount of RAP (Ci) in NPs were determined by HPLC as mentioned in the previous section. The released percentage was calculated according to the equation (3).

(3) $$\mathrm{Released}\;\mathrm{percentage}\;\left(\%\right)=\left(\mathrm{Ci}‒\mathrm{Cr}\right)/\mathrm{Ci}\times 100$$

### Cell culture and proliferation assay

Human prostate cancer cell line PC3 was used to evaluate the *in vitro* proliferation inhibition effect of RAP-loaded NPs. PC3 cells were maintained in RPMI 1640 (Invitrogen, USA) supplemented with 10% fetal bovine serum (Gibico) and 1% penicillin-streptomycin at 37°C in humidified environment of 5% CO_2_. The medium was replenished every other day and the cells were subcultured after reached confluence.

For measurement of proliferation, cells were seeded in triplicate at 2 × 10^3^ cells/well in 96-well culture plates and incubated overnight to allow cell attachment. To evaluate the cytotoxicity of NPs, cells were treated with empty PHBHHx NPs and PHBHHxPEG NPs with the concentration of 100, 500 and 1000 μg/mL for 24 hours. Cells were incubated with the RAP-loaded PHBHHx NPs, PHBHHxPEG NPs or free RAP for 24 and 48 hours to analyze the anti-proliferation effects of RAP-loaded NPs on PC3 cell lines. Cell viability was quantified by a 3-(4,5-dimethylthiazol-2-yl)-2,5-diphenyltetrazolium bromide (MTT) assay. The absorbance of viable cells was measured at 570 nm using a microplate reader (Anthos 2020, Anthos Labtech Instruments, Wals., Austria). Relative cell viability was calculated with regard to that of the cell control at 24 hour, which was set to 100% viability.

### Cellular uptake of nanoparticles

Human prostate cancer cell line PC3 and a murine macrophage cell line RAW264.7 cells were used to investigate the *in vitro* endocytosis of NPs. Cells were seeded in 96-well plates at 1 × 10^4^ cells/well and incubated at 37°C with 5% CO_2_ in a humidified incubator overnight to allow cell attachment, followed by treating with rhodamine-loaded NPs (100 μg/mL) and free rhodamine B (with equal fluorescence intensity) of for 3 h. Medium was then discarded and cells were washed with PBS twice to remove the extracellular rhodamine B and rhodamine B-loaded NPs. New medium was added to each cell cultures again and PC3 and RAW264.7 cells were observed by fluorescence microscopy to evaluated cellular uptake of various NPs. The intracellular fluorescence intensities were quantified by fluorescence spectrophotometer (Infinite M200 PRO, Tecan Group Ltd. Switzerland).

### mTOR inhibition assay and western blot analysis

Cells were seeded in 6-well plates at 2 × 10^5^ cells/well and incubated at 37°C with 5% CO_2_ in a humidified incubator overnight to get attached and then treated with free RAP or RAP-loaded NPs for 24 h. After washing with cold PBS for two times, cells were lysated by adding RIPA lysis Buffer (50 mM Tris–HCl (pH 7.4), 150 mM NaCl, 1% NP-40), followed by incubating at 4°C for 30 min. Cell lysates were centrifuged at 10000 rpm for 5 min and supernatant was then loaded into 10% SDS–PAGE gels. After electrophoresis, proteins were transferred onto PVDF membranes, which were then blocked with 5% bovine serum albumin in TBST buffer for 1 h at room temperature. PVDF membranes were incubated overnight with an anti-phospho-p70S6 Kinase (Thr 389) antibody (Cell Signaling Technology Inc., USA, #9205), anti-p70S6 Kinase antibody (Cell Signaling Technology Inc., USA, #9202), anti-mTOR antibody (Cell Signaling Technology Inc., USA, #2972) and anti-β-actin antibody from Beijing Biosynthesis Biotechnology (Beijing, China), respectively, and subsequently hybridized with secondary HRP conjugated anti-rabbit IgGs (Zhongshan Goldenbridge Biotechnology, Beijing, China, #ZB-2301). The hybrid signals were detected by incubating with an enhanced chemiluminescence solution (ECL) purchased from Thermo scientific (#LK151361). The amount of phospho-p70S6K, total p70S6K, total mTOR and β-actin were analyzed by Quantity One software (Bio-Rad laboratory Inc., USA) and the relative phospho-p70S6K level was normalized according to the amount of total p70S6K.

### Statistical analysis

All results were expressed as mean ± standard deviation of at least three independent experiments performed in triplicates. Statistical analysis was performed using a one-way analysis of variance (ANOVA) with Bonferroni post-hoctest. Differences were considered to be statistically significant at a level of p < 0.05.

## Competing interests

The authors declare that they have no competing interests.

## Authors' contributions

XYL designed and conducted all experiments and prepared the manuscript. MCL prepared the copolymer materials and fabricated the nanoparticles. XLZ and FF participated to characterize the nanoparticles and analyzed the in vitro release profile of rapamycin. LLW carried out the cell culture and proliferation assay as well as the western blot assay of mTOR inhibition. JJM helped to analyze the data and also contributed to the manuscript preparation. All authors read and approved the final manuscript.
